# Impact of Mobile Game Addiction Tendency on Chinese University Students: A Hierarchical Linear Modeling Study

**DOI:** 10.3389/fpsyg.2022.937446

**Published:** 2022-07-04

**Authors:** Yongtao Gan, Tian Zhang, Jiahao Zhang, Xia Wu, Mengjun Shao

**Affiliations:** ^1^School of Law, Higher Education Research Institution, Shantou University, Shantou, China; ^2^Jing Hengyi School of Education, Hangzhou Normal University, Hangzhou, China

**Keywords:** mobile game addiction, influencing factors, college students internet addiction, positive emotions, negative emotions, loneliness

## Abstract

This study used hierarchical linear modeling (HLM) to investigate the differences in mobile game addiction tendencies among universities (i.e., double first-class universities, ordinary colleges and universities, and higher vocational colleges), and to examine the influencing factors of mobile game addiction tendency at the individual and university levels. The participants of this study were 4,024 college students who completed the Chinese Mobile Phone Game Addiction Scale, UCLA Loneliness Scale, and Positive and Negative Affect Scale. Loneliness (*β* = 0.052), positive emotions (*β* = −0.126), negative emotions (*β* = 0.232), and double first-class universities (*γ* = 0.368) significantly predicted mobile game addiction tendencies. A significant cross-level interaction between double first-class universities and other factors (i.e., positive emotions, negative emotions, and mobile game addiction) was observed. The novelty of this study is that it distinguishes the various effects of mobile phone addiction tendency at the individual and university levels.

## Introduction

In 2021, at almost 912 million, China had more mobile users than any other country, with approximately 1.63 billion mobile phones registered for subscriptions (Statista-China, 2021). More specifically, one of the most rapidly growing and largest groups that own and use mobile phones is comprised of young adults (18–22 years, Statista-China, 2020). Young people today, known as the “wired generation,” have been overusing their mobile devices. Consequently, their academic, family, and social lives have been negatively affected (Gökçearslan et al., 2016). Therefore, it is important to study mobile phone usage among college students.

Mobile phone overuse has adverse effects on habits and health (Lepp et al., 2014). It has decreased in-person interactions (Matthews et al., 2009) and increased the prevalence of physical and mental illnesses such as depression and sleep disorders (Beranuy et al., 2009; Thomée et al., 2011); however, the most direct negative consequence has been phone addiction (Takao et al., 2009; Chóliz, 2010). In recent years, the literature on mobile phone addiction has expanded, primarily delving into the reality of mobile phone addiction (Billieux et al., 2015), its precursors (Walsh et al., 2011; Hong et al., 2012), as well as its effects on people’s health (Thomée et al., 2011), academic progress (Lepp et al., 2014), behaviors (Wang et al., 2014), mental health (Çağan et al., 2014), and recreation (Lepp et al., 2015). Some of the causes include the necessity to use mobile phones for entertainment (movies and games), information access, and communication.

The emergence of increasingly addictive online games (Toker and Baturay, 2021) has made mobile phone game addiction a common phenomenon. The present study aimed to investigate mobile game addiction among college students and its related influencing factors, in order to develop reasonable intervention countermeasures for preventing mobile game addiction among college students.

## Literature Review

Mobile game applications are used at a high frequency, second only to chatting platforms. “Addiction” is defined as a ritualistic, inextricable, and biological state of mind, comprising various forms such as compulsive gambling, exercise addiction, Internet addiction, gaming addiction, and so on. Generally, a phenomenon may be termed an “addiction” when an individual reaches a state of physical and psychological dependence that cannot be extricated or controlled. The same is true for mobile gaming addiction.

The main manifestations of Internet addiction are out-of-control working hours, interactive gaming addictions, compulsive behaviors, withdrawal symptoms, and related Internet-dependence symptoms (Young, 2006). Recent studies have shown that mobile online game addiction, as a new type of online game addiction, has aroused people’s attention and has been studied as an independent object; this addiction has a significant positive correlation with loneliness, anxiety, and depression (Haberlin and Atkin, 2022). Researchers have suggested that rejection, denial, severe punishment, as well as lack of emotion, understanding, and communication are important causes for college students’ addiction to online games. Students with family discord or breakdown are more likely to find a sense of belonging and satisfaction in online games (Han et al., 2018).

Lopata (1969) defined loneliness as an emotional state of yearning for companionship that differs from the person’s current reality. In recent years, it has been associated with addiction to mobile phone use. Moreover, loneliness refers to a negative state of mind caused by subjective dissatisfaction with human relationships and the possibility of depression or negativity (Schmidt and Sermat, 1983). Using mobile games for a long time may lead to loneliness. People with an Internet addiction might feel lonely or irritable when restricted from using the Internet, just as those with a gambling or drug addiction (Wang et al., 2015). The main activities involved in Internet addiction are gaming, porn, online chatting, grid live, and collecting information. Nowadays, many people use network communication and games to try and combat loneliness, and researchers have pointed out that people can alleviate their offline loneliness through online self-disclosure and recreational Internet use (Lopez-Fernandez et al., 2018).

Emotion is the product of the interaction between individuals and the environment, and that positive and negative emotions are both sides of the same coin, which are determined by the results of events, situations, personal assessment, and processing (Lazarus, 1991). Emotions consistent with individual goals are positive emotions, whereas those inconsistent with goals are negative emotions. With the popularity of mobile games and their unique interaction patterns, college students often indulge in pathological Internet use to escape obstacles or discomfort (Hong et al., 2012). The particularity of the development and expression of college students’ emotions, and the limitations of emotion management and expression channels, make it easy for college students to indulge in the Internet to escape their negative emotions (Han et al., 2018). Negative emotion is one of the factors influencing mobile phone addiction. In this context, the academic community has been paying significant attention to the association between mobile phone addiction and interpersonal issues (Seo et al., 2016; Troussas et al., 2022), depressive state (Thomée et al., 2011; Wang et al., 2014, 2019; Coyne et al., 2019), and anxiety disorders (Beranuy et al., 2009; Hong et al., 2012; Liu et al., 2017). In relevant empirical studies, Cheever et al. (2014) also found that social anxiety was positively correlated with mobile games to a high degree.

Beyond accessing information, majority of online users utilize their mobile phones to interact with others, socialize, enjoy entertainment, explore new domains and knowledge, accomplish work-related projects, perform inquiries, and so on (Papakostas et al., 2021; Troussas et al., 2022). These users experience positive emotions, such as satisfaction and enjoyment, through the improved capabilities of their gadgets. Considering the enjoyable experience that this technology offers, such emotional and positive reinforcements could lead to their continuous or extreme use (Longstreet et al., 2019, 2021). However, the underlying relationship between negative emotions, positive emotions, and mobile phone addiction should also be considered.

Based on the above studies, there may be a correlation between loneliness, positive/negative emotions, and the tendency toward mobile game addiction. At the university level, there are also significant differences in mobile game addiction (Troussas et al., 2020). While previous studies reported the negative consequences of mobile game addiction, they failed to address the prevalence of this issue among various university configurations (Krouska et al., 2020). Hence, this paper attempts to perform an analysis to identify the considerable differences, if any, among the modes of deviance in the university context.

In previous studies, ordinary least squares (OLS) was used to analyze the influencing factors of mobile game use at the individual level. However, college students are not a single individual, and they are bound to be affected by the organizational variables of their university. Hierarchical linear modeling (HLM) was adopted to examine whether the data had a nested or hierarchical structure, with the former clearly exemplified in a multilevel model with distinct variances established for every level. Additionally, postulating individual student-level regressions is possible for each university. In this case, we could include the characteristics of the universities and students, and the estimates of the regression coefficients and standard errors of means showed no bias. Therefore, a two-level linear model was established in this study.

Despite the considerable amount of studies on the common social problem of mobile phone addiction tendency, loneliness and positive/negative emotions, as well as the growing discussion on university type, prior studies have not fully addressed the intersecting topics. Therefore, the present study employed HLM to investigate the differences in mobile game addiction tendencies among university students, and to explore the impact of several factors (i.e., loneliness, positive/negative emotions) on mobile game addiction tendency at the individual and university levels.

In accordance with previous research, loneliness and positive/negative emotions would predict mobile phone addiction tendency; thus, this study developed the following research hypotheses:

*Hypothesis 1 (H1):* Loneliness positively predicts mobile phone addiction tendency among college students;

*Hypothesis 2 (H2):* Positive/negative emotions predict mobile phone addiction tendency among college students;

*Hypothesis 3 (H3):* University type cross-level moderates the associations between loneliness and mobile phone addiction tendency among college students;

*Hypothesis 4 (H4):* University type cross-level moderates the associations between positive/negative emotions and mobile phone addiction tendency among college students.

**Figure 1:**
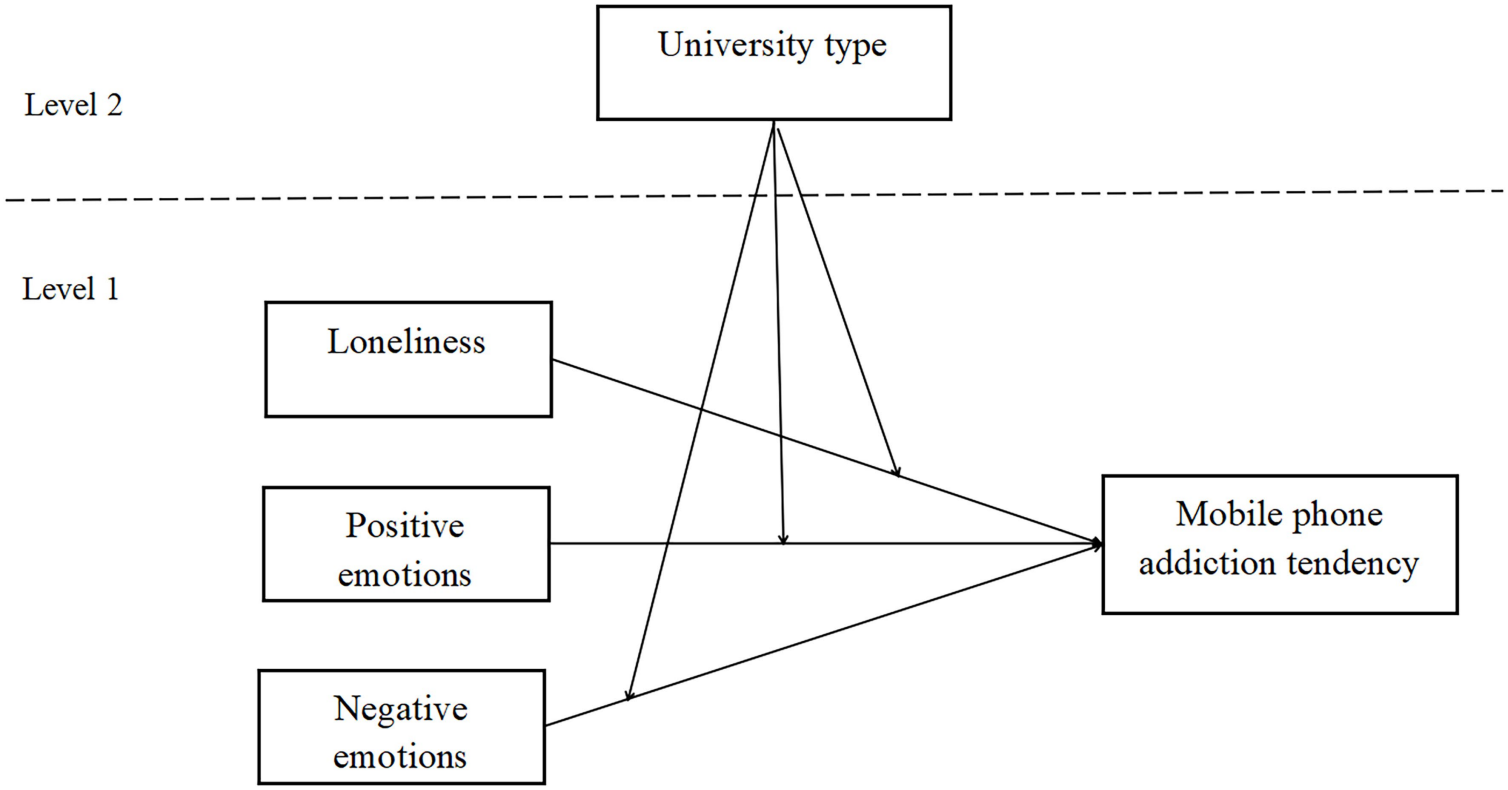
Concept model.

In light of the literature reviews and hypotheses, this study presented a theoretical model, which is presented in Figure 1.

## Materials and Methods

### Data Collection and Sample

The samples were selected with reference to the university type, grade structure, professional structure, and gender structure of higher education in China. In total, 4,024 student participants completed the questionnaires.

The research was approved by the ethical committee at the university of the first author, and the college students’ consent was obtained. Participation in the study was voluntary and anonymous.

Data were collected online between January 2021 and April 2021. Universities in the Hubei, Guangdong, Guangxi, and Shandong Provinces were sent an invitation *via* e-mail, which included information on the study purpose and a link to an online survey. Of all universities contacted, 22 responded and agreed to enroll their students in the study. Several social media platforms were used to collect as diverse a sample as possible. Prior to conducting the survey, an information sheet describing the study was provided to all potential participants. Participants were asked to complete an informed consent form if they chose to participate. After receiving the consent form, the participants were directed to the online survey. On average, it took participants approximately 20–25 min to complete the survey. To ensure valid results, we imposed the following inclusion criteria for participants: (1) they should be currently studying in colleges and universities, (2) the same IP address could only respond to the questionnaire once, to avoid repetition/redundant data, and (3) All questions administered in the questionnaires were completed. If the participant did not play mobile games, their data were excluded from our analyses. Among other measures, student participants completed instruments for the assessment of their loneliness, positive and negative emotions, and some demographic and contextual information (e.g., university type).

Table 1 shows the demographic data of the sample and the population. The former seems to be more or less descriptive of the selected universities.

**Table 1:** Sample demographics.

Item	Variable	Description	Frequency	Percentage (%)
Male	Male	0 = female, 1 = yes	1,676	41.7
Grade	Freshman	0 = no, 1 = yes	2,044	50.8
	Sophomore	0 = no, 1 = yes	611	15.2
	Junior	0 = no, 1 = yes	974	24.2
	Senior	0 = no, 1 = yes	395	9.8
Rural	Rural	0 = Urban, 1 = Rural	2,051	51.0
Two-parent family	Two-parent family	0 = other, 1 = Two-parent family	3,383	84.1
Only one child	Only one child	0 = no, 1 = yes	1,656	41.2
Discipline	Science	0 = no, 1 = yes	895	22.2
	Engineering	0 = no, 1 = yes	1,308	32.5
	Arts	0 = no, 1 = yes	223	5.5
	Humanities and Social Science	0 = no, 1 = yes	1,598	39.7
University type	Double first-class universities	0 = no, 1 = yes	687	17.1
	Ordinary colleges and universities	0 = no, 1 = yes	2,664	66.2
	Higher vocational colleges	0 = no, 1 = yes	673	16.7

### Dependent Variables

#### Chinese Mobile Phone Game Addiction Scale

Chinese Mobile Phone Game Addiction Scale is adapted from the mobile phone addiction index developed by Leung (2008). The CMPGA includes 17 items and assesses four aspects of mobile phone addiction: (1) control craving, (2) anxiety and feeling lost, (3) withdrawal and escape, and (4) productivity loss; it uses a 5-point Likert scale ranging from 1 (completely disagree) to 5 (completely agree), with higher scores indicating higher levels of mobile game addiction. The Cronbach’s internal consistency reliability of the CMPGA was 0.85.

### Independent Variables

#### UCLA Loneliness Scale

UCLA Loneliness Scale (Version 3) has been widely applied to assess loneliness (Russell, 1996). The scale is one-dimensional, containing 20 items, 11 negative (lonely) terms, and 9 positive (not lonely) terms; the Cronbach’s internal consistency reliability of this scale was 0.88.

#### Positive and Negative Affect Scale

Watson et al. (1988) developed PANAS based on a two-dimensional structure of emotion. Following the introduction, all participants completed an emotional report form, where they rated themselves on a 4-point Likert-type scale (“1 = not at all” to “4 = extremely”) by evaluating the degree to which they experienced particular emotions (amusement, anger, anxiety, disgust, energy, industriousness, enthusiasm, determination, attentiveness, dynamism, restlessness, hostility, irritability, nervousness, fidgeting, fearfulness, engagement, joy, interest, and sadness). We used confirmatory factor analysis to test for positive emotions (PE) and negative emotions (NE). The Cronbach’s internal consistency reliability of the scale was 0.80 for PE and 0.87 for NE, indicating good reliability.

### Hierarchical Linear Modeling Analysis

The HLM comprises at least two linear regression equations. At every level of the data structure, such equations may include explanatory variables. For instance, a regression equation exists at the lower level (level 1) for every university, which links the mobile gaming behavior of students to at least one of their attributes (e.g., gender, family type, and living area). From one university to another, the association of mobile game use with such attributes, along with positive or negative emotions and loneliness, differs accordingly, and is denoted by a set of regression coefficients. Subsequently, at least one characteristic of the university, such as region or type, predicts an institution’s set of regression coefficients at a higher level (level 2).

An HLM-based investigation produces a decomposition of the total variance between inter-university, intra-university, and student components. Given this requirement for data that comes from nested environments, this study utilized HLM for regression analysis rather than traditional OLS or logistic regressions. The level 1 regression demonstrates how much the variations in student attributes, negative/positive emotions and degree of loneliness, account for the differences in mobile game addiction tendency among students within various universities, specifically the first component. Likewise, a specific level 2 regression output expresses how much the differences in university attributes, such as the designation of the double first-class, ordinary, and higher vocational colleges, could account for the variations in university means or adjusted university means (i.e., the second component). In essence, HLM is appropriate for probing the differences in mobile game use among various university types.

This study utilized the HLM7 software to perform complex computations related to HLMS fitting. It is intended to manage the data structure that includes plausible values for every student evaluated. Running once for every set of these values is the selected method of analysis for each model. In this case, such values are dependent variables in the regression for each run. According to Mislevy et al. (1992), the averages of the findings are the concluding estimates.

The final standard errors were derived in accordance with the standard sample data procedures. It then merged two estimates: (a) measurement error generated from the variations in the findings across the series of plausible values and (b) sampling variability based on the initial set of these plausible values. This process was automated using the HLM program.

The analysis was conducted in two phases. In the first phase, the descriptive statistics and intercorrelations of the variables were presented. In the second phase, HLMs were employed to examine whether university types could significantly influence the dependent variables after correcting for the nesting structure and possible autocorrelation. Furthermore, numerous models were examined. First, a null model was developed; second, only loneliness, positive/negative emotions, and the dependent variable were added to the model; and third, level 1 predictors, including demographic variables and the three factors, as well as the university type were added to the model. To discern whether the effects of university type were significant, university type and the dependent variables were included, and only those verified as significantly affected by university type were selected for further analysis. In addition to the direct impact on the dependent variable and university type after the process added cross-level interactions, the study aimed to determine whether university type moderated the relationship between the other predictors in the model. Finally, to compare the differences with previous models, the data were used for OLS.

## Results

The descriptive statistics and intercorrelations of the variables are presented in Table 2. The zero-order correlations were consistent with expectations, mobile game addiction tendency was positively associated with negative emotions, and mobile game addiction tendency was negatively associated with positive emotions. Positive emotions were negatively associated with negative emotions.

**Table 2:** Descriptive statistics and correlations (*N* = 4,024).

Variable	1	2	3	4
1. Mobile Game Addiction Tendency	1.000			
2. Loneliness	0.249^**^	1.000		
3. Positive emotions	−0.109^**^	−0.403^**^	1.000	
4. Negative emotions	0.308^**^	0.358^**^	−0.060^**^	1.000
*M*	33.68	43.71	32.3	24.23
SD	13.38	10.576	7.798	7.972

** *p < 0.01*.

The null model was used to test whether there were significant differences in the dependent variable (mobile game addiction tendency) between the groups (Table 3). The results demonstrated the residual error of mobile game addiction tendency within the group (level 1) *σ* ^2^ = 0.338 and the inter-group variance *τ*_00_ = 0.369 (*p* < 0. 001), ICC (1) = τ_00_/(τ_00_ + σ^2^) = 0.5219. This indicates that 52.19% of the total variation in mobile game addiction tendency was caused by the difference between universities, which required cross-level data analysis.

**Table 3:** HLM regressions predicting mobile game addiction tendency.

Fixed effects	Null Model	Model 1	Model 2	Model 3	OLS
**Level-1**					
Loneliness		0.055^***^	0.054^***^	0.052^***^	0.065^***^
Positive emotions		−0.009^**^	−0.009^**^	−0.126^**^	−0.038^*^
Negative emotions		0.198^***^	0.197^***^	0.232^***^	0.179^***^
Two-parent families			0.023	0.027	0.086^**^
Male			0.260^***^	0.260^***^	0.288^***^
Rural area			0.023	0.032	0.03
Only one child			0.046^**^	0.031	0.002
Sophomore			0.094^**^	0.097^**^	0.001
Junior			0.100^**^	0.102^**^	0.267^***^
Senior			0.173^**^	0.146^**^	0.014
Science			0.062	0.059	0.096
Engineering			0.137^***^	0.087^*^	0.079^**^
Arts			0.080	0.087^*^	0.276^***^
**Level-2**					
Double first-class universities			0.460^**^	0.368^**^	0.603^*^
Higher vocational colleges			0.032	0.020	0.067
Double first-class universities ^*^ loneliness				0.018	−0.058
Double first-class universities^*^ positive emotions				−0.034^**^	−0.321^***^
Double first-class universities ^*^ negative emotions				0.199^**^	0.341^***^
**Random effects**					
**Level-1**					
The residual error, σ^2^	0.338	0.303	0.303	0.294	
**Level-2**					
The intercept, u_0_	0.369^***^	0.290^***^	0.258^***^	0.253^**^	
-2LogLikelihood	7252.10	6835.87	6823.24	6573.34	

Grade category dummy variables were included in these models, with Freshman omitted as the reference category; Discipline category dummy variables were included in these models, with humanities and social science omitted as the reference category; university type category dummy variables were included in these models, with ordinary colleges and universities omitted as the reference category. They were removed from the tables to conserve space. No. of observations = 4,240. Standardized coefficients with standard errors are in parentheses. ^*^p < 0.05, ^**^p < 0.01 and ^***^p < 0.001.

Model 1 showed results from the random model and indicated that loneliness (*β*_1_ = 0.055), positive emotions (*β*_2_ = −0.009), and negative emotions (*β*_3_ = 0.198) were significant predictors of the mobile game addiction tendency.

Model 2 introduced various independent variables from the intercept model at level 1 and level 2. As expected, loneliness (*β* = 0.054) and positive (*β* = −0.009) and negative emotions (*β* = 0.197) were all significant predictors of mobile game addiction tendency at level 1 after controlling for demographic variables. Two-parent families, rural areas, science and arts, by contrast, seemed unrelated to the indicator. Furthermore, only double first-class universities positively predicted the dependent variable, suggesting an interaction effect with the university type or, after controlling for the recently incorporated level 1 variables, a loss of significance. In the following model, these issues were addressed and clarified.

After adding cross-level interaction effects, the third HLM model showed minimal changes. Loneliness (*β* = 0.052), positive emotions (*β* = −0.126), negative emotions (*β* = 0.232), and double first-class universities (*γ* = 0.368) remained significant, and their respective interaction effects indicated some significance. Therefore, H1, H2, and H4 were confirmed. This finding indicated that these variables were related to mobile game addiction tendency, and the university environment had a significant effect on the strength of that relationship. The significance of university-type cross-level interactions was interesting; double first-class universities were found to significantly moderate the relationship between positive/negative emotions and the dependent variable, after controlling for other variables. The interaction effects were significant even after controlling for all other effects. In terms of the interaction effect, double first class * positive mood (*γ* = −0.034) and double first class * negative mood (*γ* = 0.199) were significant interaction effects. The results demonstrated the existence of moderating effects between positive emotion and mobile game addiction, negative emotion, and mobile game addiction in double first-class universities.

Therefore, it can be said that the increase in mobile game use due to positive/negative emotions is significant only in double first-class universities, and not in higher vocational colleges, ordinary colleges, or universities; moreover, higher positive/negative emotions were directly associated with lower mobile games, and double first-class universities moderated the relationship.

In this study, traditional OLS and HLM methods were used to detect the results. The coefficients of the HLM slope model and OLS model differed significantly. The OLS model equates organization-level factors with individual-level factors; hence, it is possible to have upward or downward errors in the coefficients of university variables. In addition, in this study, the OLS method did not consider the differences between different colleges and universities in detecting the influence of individual characteristics on college students’ mobile game addiction tendencies. As a result, the differences at the university level were identified as the role of individual factors, without distinguishing parameter variation from sampling variation. For example, for junior students, the coefficient estimated by the OLS method was higher than that estimated by the HLM method (0.267 vs. 0.102). A possible reason for this deviation is that junior students are graduates from vocational colleges, while those from ordinary universities and double first-class universities are intermediate students. The OLS method could not control the correlation between university types, so the role of mobile game addiction in junior students was overestimated. Although the coefficients were different between the OLS and HLM models, the results showed that the score of mobile game addiction tendency among art students was the highest. Art students usually have more leisure time, and they pay less attention to academics; by contrast, the addiction tendency to mobile games among engineering students was significantly lower than that of other disciplines, as they have heavier learning tasks and need to spend more time on their studies.

## Discussion

This study used HLM7 software to perform complex computations related to HLMs fitting in order to investigate the cross-level moderating effects of university type on the association of loneliness with mobile phone addiction tendency, as well as on the association of positive/negative emotions with mobile phone addiction tendency. The findings showed that positive emotions were significantly negatively correlated with mobile game addiction, while loneliness and negative emotions had a significant positive effect on the use of mobile games, which is in line with the direction of the research hypotheses, that is, H1, H2, and H4 were verified. The strengths of the study include the analysis of multiple data points for most participants, the use of a well-validated instrument that provides a comprehensive assessment of mobile game addiction, and sophisticated statistical analyses.

This study tried to establish a cross-level moderated model examine the influencing mechanism of mobile game addiction tendency. Although prior studies on the associations between mobile game addiction tendency and various variables in different contexts have been made, few studies concentrated on the cross-level impact of mobile game addiction tendency in Chinese universities. Therefore, this study has enriched previous studies. The findings of this research are also consistent with those of previous studies (Lee and Kim, 2019; Kristensen et al., 2020; Krouska et al., 2020). Distinguishing between the different roles of mobile phone addiction at the individual and university levels is an important novelty of this study. The cross-level moderated model between university type and mobile game addiction tendency are very complex, because it has three cross-level moderating effects, two direct impact at university level and three direct impact at individual level. Examining the cross-level moderating model between those variables is momentous for comprehending the influential mechanism of mobile game addiction tendency.

### General Discussion

The score of mobile game addiction tendency in this study was not high (M = 33.68), but there was a significant difference between those with the highest score (85 points) and the lowest score (17 points) in the mobile game addiction scale. Although the overall tendency of the whole sample to become addicted to mobile games is not high, some of the study samples belong to a group with a high risk of addiction to mobile games.

According to cognitive-behavioral theories, people’s behaviors affect their emotions and cognition, and vice versa (Xu et al., 2021). Hence, maladaptive behavior, such as mobile phone addiction, could impact a person’s feelings and perceptions. In the context of negative emotions, recent studies have shown that the degree of mobile phone addiction is a significant positive predictor. Furthermore, Özparlak and Karakaya (2020) discovered that Internet addiction adversely impacts the physical, psychosocial, and mental health of adolescents.

The results of this study showed that students with a higher degree of loneliness scored higher on mobile game addiction. Individuals with addiction tendencies in mobile games showed high loneliness, and correlation analysis also showed that mobile phone addiction was positively correlated with loneliness (Choi et al., 2015; Aker et al., 2017; Kim et al., 2017). Mobile game addiction usually aims to obtain psychological compensation in the virtual game world, in order to escape from loneliness, depression, or annoyance in one’s reality. Individuals who indulge in mobile games for a long time and become excessively addicted might turn down social opportunities. With the decrease in social communication activities, interpersonal emotions become increasingly indifferent and distant, and finally, individuals experience loneliness. This is consistent with the findings of recent studies on technology addiction (Pourrazavi et al., 2014). Mobile games have become an escape from loneliness. Over time, the degree of mobile game addiction will increase, and the cycle of addiction will continue.

The results indicated that positive and negative emotions were correlated with mobile game addiction tendencies. Mobile phones feature functions similar to computers and the Internet. As a result, outcomes related to mobile phone addiction could be similar to those of Internet addiction, particularly negative emotions and negative social interactions. With reference to the interpersonal difficulties, this study hypothesized that neglecting in-person interactions due to phone addiction could give rise to relationship conflicts, leading to intensified negative emotions such as loneliness, social phobia, and depression (Egger and Rauterberg, 1996; Gong et al., 2019). Furthermore, many characteristics of the Internet, such as accessibility to the physical environment, anonymity of activity content, and immediacy of connection speed, make it easy for college students to excessively or inappropriately use online games to reduce or avoid negative emotions caused by life events or social interactions. (Young, 2006).

Using the cross-level interaction model affected the individual-level factors with university-level distinguishing differences, and our study found that double first-class university college students have a higher tendency for loneliness, negative emotions, and mobile game addiction, while double first-class universities moderate the correlation between positive/negative emotions and mobile game use. Findings from the “2018 Chinese university student tracking survey (PSCUS)” of the Chinese Academy of Social Sciences showed that more than 60% of the students in double first-class universities played games (China Youth News, 2019). Some students in double first-class universities had been “homestead men” and “homestead women” since childhood. In addition to learning, they spend their leisure time using electronic products. Before attending university, due to heavy learning tasks, the time spent playing games was strictly controlled by their parents. However, while at university, learning tasks became relatively easy. In addition, the teachers were not usually strict, and many students staying in the dormitory played games, including during the night (China Youth Daily, 2019). Students in double-class universities also face greater academic pressure, are surrounded by students who are equally competent, and this sense of competition can make them choose to study harder. This sense of competition makes them feel lonely When faced with greater competition and frustration, they are more inclined to experience negative emotions, which can be explained by the fact that students in double first-class universities have more stress in terms of studies and less social activities, which leads them to depend more on mobile phone games to relieve stress (Kircaburun et al., 2020).

Additionally, at the individual level, this study paid special attention to gender influences on mobile phone game addiction, which showed significant gender differences and significant differences between students of different majors. The results of this study showed that art students have significantly higher mobile phone game addiction than other disciplines, which may be explained by the fact that students of art disciplines usually have more leisure time. Meanwhile, other researchers established that low-income adolescents had significantly higher likelihood of using their mobile phones for Internet usage (Lin and Liu, 2020). This shows that the level of economic development also leads to differences in the propensity to be addicted to mobile phone games.

In summary, the results imply that there are significant differences in the mobile game addiction tendency of university students, depending on their degree of loneliness, positive/negative emotions, and the type of university Furthermore, there is a significant cross-level interaction between double first-class universities and other factors (i.e., positive emotions, negative emotions and mobile game addiction) in the HLM model.

### Research Limitations and Future Suggestions

Despite its strengths, this study has several limitations. First, the data are single, self-reported opinions and behaviors, which, according to Bachman and Paternoster (2009), are usually beneficial for measures of deviance but have certain limitations. More specifically, each student could interpret the questions differently. However, self-reported data have advantages that outweigh the relevant limitations. Second, regarding the dependent variables, the questions inquired about the students’ mobile game behaviors throughout their entire time of use. In this case, differences among various universities could have existed prior to their entry or admission. These behaviors are less common at earlier ages and are unlikely to be the case. However, these data do not prevent such a likelihood. Additionally, all participants were university students, because of which, the sample was not representative of the Chinese population in terms of education and socio-economic status. In general, however, the findings appeared to be consistent with a much extensive body of literature.

To address the above-mentioned limitations, future studies should conduct experiments on loneliness, positive/negative emotions, and mobile phone addiction tendency, drawing from the fields of ethology and cognitive neuroscience technology. The negative impact of mobile phone overdependence on individuals can be reduced through interventions targeting loneliness and positive/negative emotions. Finally, some claim that the phenomena recognized should be addressed, such as by classifying variables through which university configuration affects those behaviors of mobile game use or possible practical interventions.

## Data Availability Statement

The raw data supporting the conclusions of this article will be made available by the authors, without undue reservation.

## Ethics Statement

The studies involving human participants were reviewed and approved. This study was performed in line with the principles of the Declaration of Helsinki. Approval was granted by the Ethics Committee of Shaoutou University Medical College. IRB code is SUMC-2021-101. The patients/participants provided their written informed consent to participate in this study.

## Author Contributions

TZ, MS, JZ, and XW performed the material preparation, data collection, and analysis. YG wrote the first draft of the manuscript and all authors commented on previous versions of the manuscript. All authors have read and approved the final manuscript.

## Funding

This study was funded by the National Social Science (Educational Project) Foundation of China, Grant/Award Number: BMA18004. Funding body had no influence on study design, data collection, data analysis, data interpretation or writing the manuscript.

## Conflict of Interest

The authors declare that the research was conducted in the absence of any commercial or financial relationships that could be construed as a potential conflict of interest.

## Publisher’s Note

All claims expressed in this article are solely those of the authors and do not necessarily represent those of their affiliated organizations, or those of the publisher, the editors and the reviewers. Any product that may be evaluated in this article, or claim that may be made by its manufacturer, is not guaranteed or endorsed by the publisher.
